# *Bacillus subtilis* Biofilms: a Matter of Individual Choice

**DOI:** 10.1128/mBio.02339-18

**Published:** 2018-11-27

**Authors:** Bridgett K. Ryan-Payseur, Nancy E. Freitag

**Affiliations:** a Department of Microbiology and Immunology, University of Illinois at Chicago College of Medicine, Chicago, Illinois, USA

**Keywords:** *Bacillus subtilis*, SinR, YmdB, biofilm formation, bistability

## Abstract

Bacillus subtilis has the capacity to choose between two mutually exclusive lifestyles: biofilm formation and flagellum-mediated swimming motility. Interestingly, this choice is made at the individual cell level, with bacterial cells in a population expressing genes required for biofilm formation or genes required for swimming motility but not both. A bistable switch controls the biofilm-versus-swimming decision, resulting in an evolutionarily favorable strategy known as “bet hedging” that ensures that subpopulations of bacteria continue to grow as conditions change and/or become unfavorable. In a recent issue of *mBio*, J. Kampf and colleagues (mBio 9:e01464-18, 2018, https://doi.org/10.1128/mBio.01464-18) reported the use of a combination of genetics and microfluidics to reveal that the interplay that occurs between the SinR and YmdB proteins underlies the B. subtilis choice between biofilm formation and swimming motility. Their report suggests that B. subtilis experiences selective pressure to form biofilms while maintaining reserve cell subpopulations with the capacity to swim away.

## Abstract (precis)

Bacillus subtilis has the capacity to choose between two mutually exclusive lifestyles: biofilm formation and flagellum-mediated swimming motility. Interestingly, this choice is made at the individual cell level, with bacterial cells in a population expressing genes required for biofilm formation or genes required for swimming motility but not both.

## COMMENTARY

Bacterial populations can at times be viewed as groups of genetically identical cells with individuals responding in concert to environmental cues. Coordinated multicellular bacterial behavior is exemplified by processes such as quorum sensing, a mechanism by which bacterial populations synchronize responses and regulate behavior based on cell density (1). Alternatively, despite genetic identity, individual members of a bacterial population may instead behave quite differently from their neighbors. This individualized bacterial behavior is often based on molecular switches that respond to a threshold concentration of a signaling molecule, such that activation of the switch becomes stochastic (2). Populations of the Gram-positive bacterium Bacillus subtilis form biofilms, but this behavior is bistable in that individual bacteria within the biofilm can express genes for motility or genes for biofilm formation but generally not both at the same time. These bistable B. subtilis cells are programmed to form a biofilm or to swim and thereby typify a behavior known as “bet hedging” (2, 3). Bacterial bet hedging produces a physiologically heterogenous group of genetically identical cells and improves the chances of bacterial survival should conditions become unfavorable for one group (for example, the biofilm formers) versus another (the swimmers).

Recent work by Kampf et al. (4) explored the mechanisms underlying B. subtilis bet hedging with respect to biofilm formation versus flagellum-mediated swimming motility. Biofilm formation requires the expression of gene operons that contribute to polysaccharide synthesis and deposition of amyloid fibers (5). A small DNA-binding transcription factor known as SinR is central to the determination of cell fate with respect to biofilm formation versus swimming motility (6). SinR acts as a repressor and binds to the promoters of biofilm operons *epsA-O*, *tapA-sipW-tasA*, and *slrR* to prevent transcription and expression of these genes. SinR activity is antagonized by two proteins, SinI and SlrR, which function to bind to and remove SinR from its target promoters and thereby permit the expression of biofilm genes (6). Added to this regulatory mix is the phosphodiesterase YmdB, which is somehow involved in controlling the bistable switch between biofilm and motility gene expression, including that of the flagellum protein (*hag*) and autolysin proteins (7). Biofilm genes are not expressed in the absence of YmdB, whereas motility genes are highly expressed. YmdB mutants that lack phosphodiesterase activity are unable to form biofilms, and strains of B. subtilis that have excess cyclic di-AMP are inhibited for biofilm formation; however, the physiological substrate of YmdB has yet to be identified.

Kampf and colleagues set out to better understand the role of YmdB and its relation to bistable gene expression as governed by SinR. They used a microfluidic platform to observe the switching patterns of individual cells based on reporter gene expression profiles over time and, with the addition of genetic analyses, gained insight into the relationship of YmdB and SinR and into their contributions to B. subtilis decision making.

Kampf and colleagues first verified that the loss of YmdB from B. subtilis inhibited expression of biofilm genes. Using microfluidics in combination with reporter gene constructs that included the fusion of the yellow fluorescent protein (YFP) gene to *hag*, the flagellin gene, and of the cyan fluorescent protein (CFP) gene to *tapA*, a gene involved in biofilm formation, they were able to image expression patterns over time in individual cells in both Δ*ymdB* and wild-type strains. Subpopulations of cells became readily detectable over time, with wild-type bacteria giving rise to cells expressing neither motility nor biofilm genes, cells expressing just motility genes, cells expressing just biofilm genes, and even a few cells expressing both motility and biofilm genes. The results of those experiments provide a glimpse into the dynamic variation within the population and the interconversion of different cell types over time.

As anticipated, Δ*ymdB* mutant populations did not give rise to many cells expressing biofilm-associated genes. The majority of cells expressed *hag*-CFP or expressed neither reporter gene, although cells expressing *tap*-YFP did infrequently become visible over time. Kampf et al. characterized independently isolated mutants based on biofilm restoration to the Δ*ymdB* mutant to determine how suppressor mutations compensated for the loss of YmdB and restored biofilm formation, with the ultimate goal of gaining insight into the direct targets of YmdB that influence the activity of the SinR regulator. Among 14 mutants containing suppressor mutations in *ymdB* deletion strains, 12 contained point mutations within the *sinR* gene and 2 contained deletions of *sinR* and expressed biofilm genes and motility genes simultaneously. Thus, rather than finding indications of YmdB substrates that might lead to the identification of, for example, second messenger nucleotides as targets for YmdB phosphodiesterase activity, all suppressor mutations affected the expression or activity of SinR. This finding reveals that the homeostasis of SinR is the major function of the YmdB phosphodiesterase.

While the precise role of YmdB has yet to be determined, the work by Kampf et al. clearly establishes the close ties between YmdB function and SinR activity in regulating the bistable switch between biofilm formation and motility. Biochemical analyses of five SinR suppressor mutants with single amino acid substitutions shed additional light on the functional regions of SinR and its multimeric interactions.

The phosphodiesterase activity of YmdB is important for biofilm production, and yet previous work by those authors excluded the involvement of YmdB in the hydrolysis of second messenger nucleotides (7). It is possible therefore (as speculated by those authors) that YmdB may act through direct interactions with nucleic acids, perhaps even affecting the stability and/or translation of *sinR* transcripts. This may explain why suppressor mutations that affect SinR activity, rather than phosphodiesterase target molecules, were identified in this work.

Finally, the fact that *ymdB* mutants appear to readily acquire suppressor mutations that restore biofilm formation suggests that there is selective pressure with respect to this bacterial behavior. Whatever the advantages of biofilm formation, the ability of individuals to switch and thus maintain a subpopulation of swimmers through SinR regulation provides the bet hedging that guarantees bacterial survival via the swim to freedom should conditions suddenly prove less than favorable for the more recalcitrant biofilm members.
